# Serum MAP1A as a Potential Biomarker for Autism Spectrum Disorder

**DOI:** 10.3390/brainsci16050478

**Published:** 2026-04-29

**Authors:** Jiwon Jeong, Seung Hyeon Lee, Dongsun Park

**Affiliations:** Laboratory of Veterinary Toxicology, College of Veterinary Medicine, Kangwon National University, Chuncheon 24341, Republic of Korea; yru0805@gmail.com (J.J.); seough03@naver.com (S.H.L.)

**Keywords:** autism spectrum disorder, serum biomarker, MAP1A, synaptic genes, valproic acid model, RNA sequencing

## Abstract

**Highlights:**

**What are the main findings?**

- Synapse-related genes dysregulated in an ASD model brain were systematically screened and cross-validated with human serum proteomic data.
- MAP1A was identified as a translational blood-based biomarker candidate showing consistent alteration across animal and human datasets.

**What are the implications of the main findings?**

- These findings suggest the feasibility of using peripheral blood biomarkers for the objective diagnosis of autism spectrum disorder.
- The cross-species brain-to-blood validation strategy provides a practical framework for discovering clinically translatable ASD biomarkers.

**Abstract:**

**Background/Objectives**: Autism spectrum disorder (ASD) is a heterogeneous neurodevelopmental condition currently diagnosed through subjective behavioral assessments. Objective blood-based biomarkers are needed to enable earlier and more accurate identification. In this study, we aimed to identify synapse-related biomarkers associated with ASD and evaluate their potential as serum-based indicators. **Methods**: RNA sequencing was performed on the cerebellum, hippocampus, and cerebral cortex of a valproic acid-induced rat model of ASD to identify differentially expressed genes (DEGs). Functional enrichment analyses, including Gene Ontology and Kyoto Encyclopedia of Genes and Genomes, were conducted to explore associated pathways. Synapse-related hub genes were selected by comparison with the SFARI autism gene database, and the serum expression of candidate proteins was assessed using Western blotting. **Results**: A total of 692, 813, and 1059 DEGs were identified in the cerebellum, hippocampus, and cortex, respectively. Enrichment analyses highlighted dendrite development, postsynaptic density, and glutamatergic synapse pathways as significantly affected. Six synaptic hub genes were prioritized, among which serum MAP1A expression was significantly elevated in the ASD rats. **Conclusions**: These findings suggest that serum MAP1A may represent a potential biomarker reflecting synaptic abnormalities in ASD. Further validation in human cohorts and integration into a multi-marker framework are warranted to account for the heterogeneity of ASD.

## 1. Introduction

Autism spectrum disorder (ASD) is a neurodevelopmental condition characterized by impairments in social communication alongside restricted and repetitive behaviors. Although genetic and environmental factors are implicated, the exact mechanisms underlying ASD remain unclear. Currently, diagnosis relies almost entirely on behaviorally defined features, which are subjective and depend on the examiner’s interpretation [1]. Detection of this condition is often delayed when early symptoms are subtle, and comorbid conditions such as depression or sleep disorders can further complicate diagnosis. Despite the increasing prevalence of ASD, no reliable and objective diagnostic indicators have been identified [2]. Therefore, the development of reproducible biological markers for the early and accurate diagnosis of this condition is urgently needed.

Biomarkers are measurable biological indicators that can aid in diagnosing and predicting disease presence and progression. They play a critical role in clinical practice by enabling the early detection and accurate classification of disorders that are otherwise difficult to distinguish based on symptoms alone. Among these, blood-based biomarkers are particularly attractive because they are noninvasive, cost-effective, and easily measurable [3]. Such biomarkers have been successfully used to monitor neurological disorders, including amyotrophic lateral sclerosis [4]. Applying blood-based biomarkers to ASD diagnosis could increase objectivity, allow earlier detection when behavioral symptoms are less pronounced, and provide molecular insights into underlying mechanisms. Several candidates, such as elevated blood serotonin levels and inflammatory cytokines like tumor necrosis factor-α, have been proposed. However, these markers show inconsistent results across patients and fail to fully capture the heterogeneity of ASD.

Emerging evidence suggests that synaptic pruning defects are a central feature of ASD [5,6]. Increased dendritic spine density has been observed in patients with ASD [7], indicating an excess of excitatory synapses and disruption of cortico–cortical and cortico–subcortical communication. Such abnormalities contribute to excitatory/inhibitory imbalance in the neocortex, a hallmark of ASD pathophysiology, and are directly linked to core symptoms including impaired social behaviors and repetitive patterns.

Next-generation sequencing (NGS) provides an effective approach for investigating these mechanisms by identifying gene expression changes associated with synaptic pathology [8]. Moreover, if synapse-related genes identified in the brain are also detectable in blood, they may serve as objective and accessible diagnostic biomarkers. Emerging evidence indicates that central nervous system (CNS)-derived molecules can be detected in peripheral blood under both physiological and pathological conditions. Neuronal activity, synaptic remodeling, and neurodevelopmental alterations can promote the release of brain-derived proteins into the circulation through multiple mechanisms. These include increased permeability or dysfunction of the blood–brain barrier (BBB), extracellular vesicle-mediated transport (including neuron-derived exosomes), and glymphatic clearance pathways. Consistent with these mechanisms, several studies have demonstrated that neuron- and synapse-related proteins can be detected in peripheral blood and may reflect CNS pathology [3,4]

In this study, we performed an NGS analysis of brain tissue from valproic acid (VPA)-induced ASD rat models and used normal control rats for comparison. Candidate genes were further validated in blood samples to assess their potential as serum biomarkers. Identifying blood-based markers that reflect synaptic abnormalities may help overcome the limitations of behavior-based diagnosis, improve diagnostic objectivity, and contribute to the development of clinically translatable tools for ASD. This brain-to-blood validation strategy enables the identification of clinically translatable biomarkers by linking central molecular alterations to peripheral detectability.

## 2. Materials and Methods

### 2.1. Experimental Animals and ASD Model Induction

Female Sprague–Dawley rats at gestational day (GD) 11 were obtained from Daehan-Biolink (Eumseong, Republic of Korea). The animals were housed under controlled conditions (22 ± 2 °C, 55% ± 10% relative humidity, 12 h light/dark cycle) with free access to standard chow and purified water. On GD 12.5, pregnant females received a single subcutaneous injection of sodium valproate (600 mg/kg, 200 mg/mL, dissolved in physiological saline; Sigma-Aldrich (St. Louis, MI, USA), P4543-10G) to induce an autism-like phenotype in offspring. Control females received an equivalent volume of physiological saline. All females were housed individually and allowed to raise their own litters. The day of birth was designated as postnatal day (PND) 1. After weaning at PND 21, offspring were housed by sex (three to four per cage). Only male offspring were used for subsequent experiments. All procedures were approved by the Institutional Animal Care and Use Committee of Korea National University of Education (#KNUE-202402-003-02).

### 2.2. RNA Sequencing and Bioinformatic Analysis

#### 2.2.1. Sampling and RNA Isolation

At PND 42, the rats were sacrificed by decapitation, and the cerebral cortex, hippocampus, and cerebellum were rapidly dissected and frozen in liquid nitrogen. Tissues were stored at –80 °C until use. Total RNA was extracted using TRIzol reagent (Invitrogen, Carlsbad, CA, USA). The RNA concentration and purity were determined using a NanoReady F-1100 spectrophotometer (Lifereal, Shanghai, China), and RNA integrity was assessed using a Bioanalyzer 2100 (Agilent, Santa Clara, CA, USA). Only samples with an A260/280 ratio of 1.6–2.0 and RNA integrity number >7.0 were included.

#### 2.2.2. Library Preparation and Sequencing

RNA samples from the cortex, hippocampus, and cerebellum were submitted to Bioneer (Daejeon, Republic of Korea) for transcriptome sequencing. Libraries were prepared using a TruSeq Stranded mRNA Sample Prep Kit (Illumina, San Diego, CA, USA) in accordance with the manufacturer’s protocol. Paired-end sequencing was performed on an Illumina NovaSeq 6000 platform.

#### 2.2.3. Sequence Analysis

Raw FASTQ files were quality-checked and processed. Reads were aligned to the rat reference genome (Rattus norvegicus 6.0, v101) using HISAT2 [9]. Gene expression levels were quantified using HTSeq [10] and StringTie (v2.1.3b). Transcript abundance is expressed as fragments per kilobase of transcript per million mapped reads. Differentially expressed genes (DEGs) were identified with DESeq2, using thresholds of fold change > 2 and adjusted *p* ≤ 0.05. Functional enrichment of DEGs was analyzed with Gene Ontology (GO) using WebGestalt (https://www.webgestalt.org/) and Kyoto Encyclopedia of Genes and Genomes (KEGG) using ShinyGO 0.80 (http://bioinformatics.sdstate.edu/go/) (accessed on 30 September 2025). 

### 2.3. Serum Collection and Western Blot Validation

Blood samples were collected from the abdominal aorta under deep anesthesia, and serum was obtained by centrifugation at 3000 rpm for 20 min. To prepare the samples, serum was diluted 1:10 with distilled water, and 10 μL of the diluted serum was applied to each well for further analysis. Proteins were denatured at 95 °C for 5 min in 0.5 M Tris–HCl buffer (pH 6.8) containing 10% sodium dodecyl sulfate (SDS) and 10% ammonium persulfate, separated on 5% or 10% SDS–polyacrylamide gels, and transferred onto polyvinylidene difluoride membranes (25 mM Tris buffer with 15% methanol, 1% SDS, and 192 mM glycine). The membranes were blocked with 5% skim milk in Tris-buffered saline containing 0.1% Tween-20 (TBS-T; 20 mM Tris, pH 7.6; 137 mM NaCl; 0.1% Tween-20) for 1 h at room temperature and then incubated overnight at 4 °C with primary antibodies against MAP1A, CAMK2A, and GRIA1 (Table 1). After washing, the membranes were incubated for 2 h at room temperature with horseradish peroxidase-conjugated goat anti-rabbit immunoglobulin G (1:2000; Santa Cruz Biotechnology, Dallas, TX, USA). Protein bands were visualized using enhanced chemiluminescence (Pierce, Rockford, IL, USA). The Western blot analysis was conducted using three independent biological replicates per group (*n* = 3).

### 2.4. Statistical Analysis

All quantitative data are presented as mean ± standard error of the mean. Comparisons among groups were analyzed using one-way analysis of variance followed by Tukey’s post hoc test. For comparisons between two groups, an unpaired two-tailed Student’s *t*-test was used. Statistical significance was defined as *p* < 0.05. Analyses were conducted using GraphPad Prism version 9.0 (GraphPad Software, San Diego, CA, USA).

## 3. Results

### 3.1. Detection of DEGs in ASD Brain via RNA-Seq

**Figure 1:**
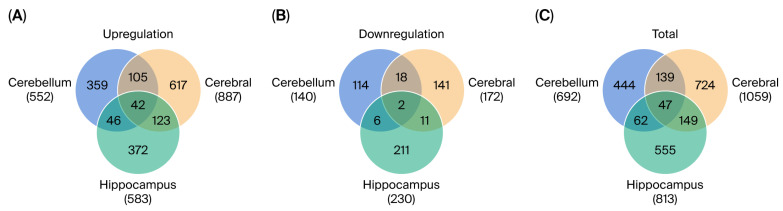
Differentially expressed genes (DEGs) identified in the brains of valproic acid (VPA)-induced autism spectrum disorder (ASD) rats. Venn diagrams showing the overlap of (**A**) upregulated DEGs and (**B**) downregulated DEGs among the cerebrum cortex, cerebellum, and hippocampus. (**C**) Combined overlap of total DEGs across the three regions.

RNA-seq analysis was performed on the cortex, hippocampus, and cerebellum of VPA-induced ASD and control rats. Differential expression analysis identified 692 DEGs in the cerebellum (552 upregulated, 140 downregulated), 813 DEGs in the hippocampus (583 upregulated, 230 downregulated), and 1059 DEGs in the cortex (887 upregulated, 172 downregulated). The overlap of DEGs among the three regions is shown in a Venn diagram (Figure 1A–C). Of the 47 DEGs common to all regions, seven overlapped with autism-associated genes listed in the SFARI database.

### 3.2. Functional and Pathway Enrichment Analyses

GO enrichment (biological process, molecular function, cellular component) and KEGG pathway analyses were performed to explore the biological mechanisms underlying ASD pathology. GO analysis revealed that the DEGs were enriched in processes related to dendrite development, postsynaptic density, dendritic spines, synapses, postsynaptic membranes, synaptic vesicle membranes, and neuronal cell bodies. The DEGs in the cortex were primarily involved in dendrite development, negative regulation of synaptic transmission, glutamatergic signaling, and neuron differentiation. The DEGs in the cerebellum were enriched in chemical synaptic transmission and negative regulation of neuron death. The DEGs in the hippocampus were associated with regulation of short-term synaptic plasticity, calcium ion response, and negative regulation of neuronal projection development.

**Figure 2:**
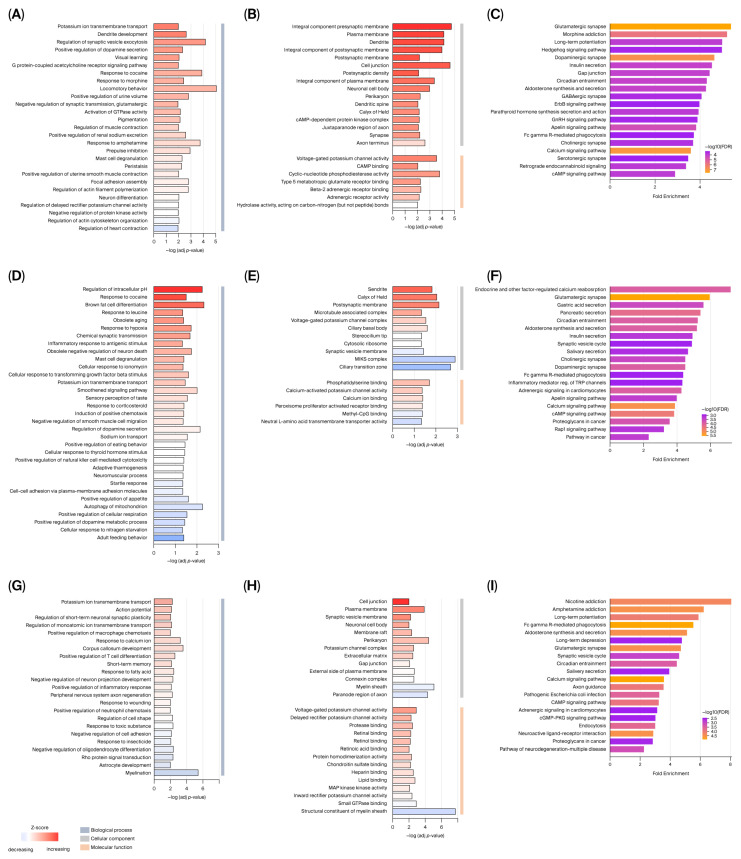
Gene Ontology (GO) and Kyoto Encyclopedia of Genes and Genomes (KEGG) enrichment analyses of differentially expressed genes (DEGs) in valproic acid (VPA)-induced autism spectrum disorder (ASD) rats. GO enrichment results for the (**A**,**B**) cerebral cortex, (**D**,**E**) cerebellum, and (**G**,**H**) hippocampus. KEGG pathway enrichment results for the (**C**) cerebral cortex, (**F**) cerebellum, and (**I**) hippocampus. Bar length represents −log10 (adjusted *p* value), and bar color indicates −log10 (false discovery rate). Fold enrichment is shown on the x-axis.

At the cellular component level, dendrites, postsynaptic membranes, synaptic vesicle membranes, and neuronal cell bodies were enriched across regions. Molecular functions included voltage-gated potassium channel activity (cortex), calcium-activated potassium channel activity (cerebellum), calcium ion binding (cerebellum), and structural constituents of the myelin sheath (hippocampus). KEGG pathway analysis indicated that the DEGs were significantly enriched in glutamatergic synapse signaling, long-term potentiation, synaptic vesicle cycling, and neurodegenerative disease pathways. Notably, glutamatergic synapse signaling was consistently enriched across all three regions (Figure 2).

### 3.3. Protein–Protein Interaction and Hub Gene Identification

**Figure 3:**
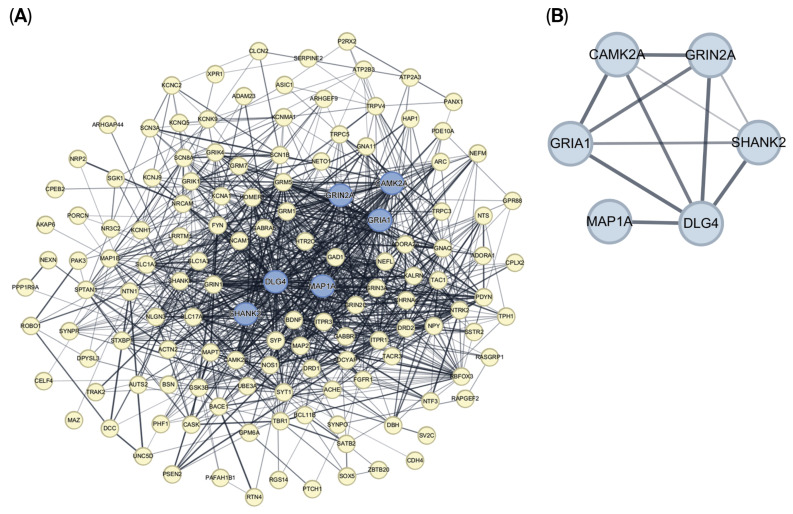
Protein–protein interaction (PPI) network and hub gene identification derived from pyramidal neuron-related DEGs. (**A**) PPI network generated from pyramidal neuron-associated DEGs (interaction score = 0.400). Node size corresponds to the degree of connectivity, and blue nodes represent hub genes. (**B**) Hub gene subnetwork highlighting six synaptic genes (GRIN2A, DLG4, GRIA1, SHANK2, CAMK2A, and MAP1A) identified using Cytoscape with the cytoHubba plugin.

Abnormalities in pyramidal neurons of the temporal lobe are frequently reported in ASD. Thus, the DEGs from the rat model were compared with pyramidal neuron-specific gene sets. A total of 150 overlapping genes were identified and subjected to protein–protein interaction (PPI) analysis using the STRING database via the NetworkAnalyst tool. The resulting PPI network contained 150 nodes and 1008 edges (Figure 3A). Hub genes were identified using the cytoHubba plugin in Cytoscape (version 3.10.1), yielding six synapse-related genes: CAMK2A, MAP1A, GRIN2A, GRIA1, SHANK2, and DLG4 (PSD95) (Figure 3B). These genes play key roles in synaptic plasticity, excitatory/inhibitory balance, and autism susceptibility (Table 2).

### 3.4. Validation of Hub Genes in Serum

**Figure 4:**
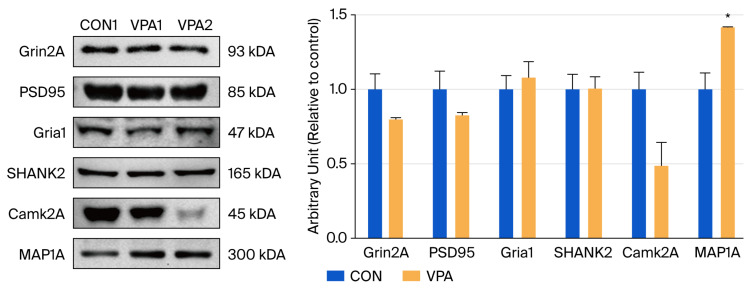
Serum validation of candidate synaptic proteins in valproic acid (VPA)-induced autism spectrum disorder (ASD) rats at postnatal day 42. Representative Western blot images (left) for GRIN2A, DLG4 (PSD95), GRIA1, SHANK2, CAMK2A, and MAP1A in serum samples from control and VPA-exposed rats. Quantitative band-intensity analysis (right) is presented as mean ± standard error of the mean (* *p* < 0.05 vs. control).

Western blot analysis was performed to examine the serum protein expression of the hub genes. MAP1A expression was significantly elevated in the serum of VPA-induced ASD rats compared with that of control rats (*p* < 0.05, Figure 4). By contrast, the serum levels of DLG4, CAMK2A, and GRIA1 were significantly decreased in the ASD group, whereas those of SHANK2 and GRIN2A showed no significant differences between the two groups. These findings highlight MAP1A as the most promising candidate as a blood-based biomarker of ASD.

## 4. Discussion

In this study, RNA sequencing was performed on the cerebellum, hippocampus, and cerebral cortex of VPA-induced ASD rats, and networks of DEGs were analyzed. Numerous genes related to synaptic structure and function were identified. GO and KEGG enrichment analyses revealed that the DEGs in the VPA-induced ASD rats were predominantly associated with dendrite development, postsynaptic density, dendritic spines, and glutamatergic synapses. Comparison with the SFARI human gene database enabled the selection of candidate proteins, among which MAP1A expression was elevated in serum, suggesting its potential as a candidate biomarker for ASD.

The prevalence of ASD has steadily increased [17], but its etiology remains largely unknown despite the identification of genetic and environmental risk factors, and effective treatments for core symptoms remain unavailable. The heterogeneity of ASD further complicates diagnosis [18]. Thus, animal models that accurately recapitulate ASD pathophysiology are essential for elucidating mechanisms and identifying biomarkers. The VPA-induced autism rat model is widely used owing to its construct validity and translational relevance [19]. Prenatal VPA exposure leads to behavioral abnormalities such as impaired social interaction and repetitive behaviors, as well as structural and functional alterations in neural connectivity [20,21]. This model replicates hallmark features of ASD, including synaptic overconnectivity and excitation/inhibition (E/I) imbalance, making it valuable for exploring mechanisms and identifying candidate biomarkers. In addition to the VPA-induced model, various animal models of ASD, including genetic models such as SHANK3, CHD8, and FMR1, have been developed to capture the heterogeneity of the disorder. Despite differences in etiology, many of these models converge on synaptic dysfunction and excitatory/inhibitory imbalance, supporting the relevance of our findings across diverse ASD models [22].

The cortex, hippocampus, and cerebellum are critical regions implicated in ASD pathology. In the cortex, abnormal pyramidal neuron connectivity and neurite overgrowth have been reported [23]. The cerebellum contributes to cognition and emotion, and reductions in cell density and synaptic connectivity have been observed [24]. The hippocampus, central to learning and memory, exhibits E/I imbalance driven by increased glutamate levels [25]. Alterations in synapse-related genes across these regions support well-established mechanisms of ASD, including neurodevelopmental abnormalities and synaptic overconnectivity.

Our RNA-seq results demonstrated that the DEGs across all three brain regions were consistently associated with synaptic pathways such as glutamatergic synapses, postsynaptic density, and dendrites, functions linked to excitatory pyramidal neurons. Pyramidal neurons, the principal excitatory neurons in the cortex and hippocampus [26], are characterized by extensive dendrites and high spine density. Excessive excitatory connectivity in pyramidal neurons can disrupt long-range cortical–cortical and cortical–subcortical communication [27], resulting in E/I imbalance [28] and altered social behaviors. Consistent with this, defective synaptic pruning and elevated spine density have been observed in patients with ASD [6,7,29]. These findings support our approach of focusing on DEGs in pyramidal neurons and cross-referencing them with SFARI human gene data, which identified six hub genes associated with synaptic plasticity. In addition, some of the identified hub genes, including GRIN2A and SHANK2, are annotated as disease-associated genes in curated databases such as OMIM, supporting their clinical relevance. This distinction highlights that the selected candidates include genes with established links to human neurodevelopmental disorders, in addition to those identified based on network-based approaches. These findings are consistent with previous RNA-seq studies in VPA-induced ASD models, which have reported dysregulation of synapse-related genes and pathways associated with excitatory/inhibitory imbalance.

MAP1A, a microtubule-associated protein essential for neurodevelopment [29], emerged as a key candidate. MAP1A stabilizes neuronal structure and contributes to dendritic remodeling by interacting with postsynaptic scaffold proteins, including PSD-93 and PSD-95 [29,30]. Deficiency of MAP1A reduces scaffold protein levels, destabilizes N-methyl-D-aspartate (NMDA) receptor complexes, and weakens excitatory synaptic currents [31]. By contrast, increased MAP1A expression enhances synaptic plasticity and may underlie the increased spine density and behavioral abnormalities observed in ASD. Prenatal VPA exposure upregulates NMDA receptor subunits and CAMK2A, thereby enhancing postsynaptic long-term potentiation [32]. Our finding of elevated MAP1A in serum, in addition to brain tissue, highlights its potential as a candidate biomarker for ASD. Although several synaptic hub genes were identified, MAP1A showed the most consistent and significant alteration in serum, supporting its prioritization in this study. However, given the heterogeneity of ASD, a multi-marker approach may provide greater diagnostic accuracy and should be explored in future studies. From a translational perspective, MAP1A may serve as a candidate blood-based biomarker for ASD diagnosis and may also have potential utility in disease monitoring. Given the heterogeneity of ASD, MAP1A is unlikely to function as a standalone marker but could be integrated into a multi-marker panel to improve diagnostic accuracy. Furthermore, its potential application in longitudinal assessment may provide additional value in tracking disease progression or treatment response.

The detection of MAP1A in serum suggests that brain-derived synaptic proteins may be transported into the peripheral circulation. Although the precise mechanisms remain to be fully elucidated, several biologically plausible pathways have been proposed. Disruption or increased permeability of the blood–brain barrier (BBB) under neurodevelopmental or pathological conditions may allow the passive leakage of neuronal proteins into the bloodstream [33]. In parallel, extracellular vesicles, including neuron-derived exosomes, have been shown to carry synaptic proteins and cross the BBB, thereby mediating brain-to-blood communication [34]. Furthermore, the glymphatic clearance system contributes to the drainage of interstitial proteins from the brain into the systemic circulation [35]. Supporting this concept, previous studies have demonstrated that CNS-derived proteins, such as neurofilament light chain and synaptic markers, can be detected in serum and serve as indicators of neuronal damage or synaptic dysfunction [3,4]. Collectively, these mechanisms support the biological plausibility of serum MAP1A as a translational biomarker reflecting central synaptic alterations.

This study has some limitations. The VPA-induced ASD model best reflects environmentally induced ASD, but only a subset of human cases is attributable to prenatal exposure [36]. Moreover, the sample size in this study was limited, reducing generalizability. MAP1A was proposed as a single biomarker, but given the heterogeneity of ASD, a multi-marker panel approach is necessary to capture the full spectrum of the disorder [37,38,39,40]. Finally, species differences necessitate further validation in human cohorts to establish clinical utility.

## 5. Conclusions

This study identified blood-based biomarkers of ASD through transcriptomic analysis of a VPA-induced rat model and serum validation of candidate proteins. Synaptic abnormalities in excitatory neurons, particularly pyramidal neurons, were identified as key pathological features of ASD. Among the identified hub genes, MAP1A expression was significantly upregulated in serum, suggesting its potential as a candidate biomarker for ASD diagnosis. These findings provide insights into the molecular pathology of ASD and support the potential for translational applications from animal models to clinical settings.

## Figures and Tables

**Table 1:** List of antibodies used in the current study.

Epitope	Company	Catalog Number	Dilution	2nd Ab (IgG)
Grin2A (165 kDa)	Abcam	ab203197	1:1000	Rabbit
PSD95 (85 kDa)	Abcam	ab18258	1:1000	Rabbit
Gria1 (47 kDa)	Abcam	ab31232	1:1000	Rabbit
SHANK2 (165 kDa)	Cell signaling	12218S	1:1000	Rabbit
Camk2A (45 kDa)	Abcam	ab52476	1:1000	Rabbit
MAP1A (300 kDa)	Abcam	ab101224	1:1000	Rabbit

2nd Ab (IgG), secondary antibody (immunoglobulin G).

**Table 2:** Hub proteins identified in protein–protein interaction (PPI) network analysis of proteins encoded by differentially expressed genes.

Gene Symbol	Description	Regulation	Degree	Biological Significance	Reference
Grin2A	Glutamate [NMDA] receptor subunit epsilon-1	Up	52	Involved in synaptic plasticity, learning and memory function	[11]
DLG4	Discs large homolog 4	Up	65	Effects on synaptic connectivity and activity	[12]
Gria1	Glutamate ionotropic receptor AMPA type subunit 1	Up	51	Long-term potentiation (LTP) induction	[13]
SHANK2	SH3 and multiple ankyrin repeat domain protein 2	Up	23	Causative genes for idiopathic autism spectrum disorders	[14]
Camk2A	Calcium/calmodulin-dependent protein kinase II alpha	Up	48	Essential roles in synaptic plasticity	[15]
MAP1A	Microtubule-associated protein 1A	Up	6	Activity-dependent dendritic modeling	[16]

## Data Availability

The data supporting the findings of this study are available from the corresponding author upon reasonable request. The data are not publicly available due to ethical restrictions related to animal experimental data.

## Abbreviations

The following abbreviations are used in this manuscript:

ASD	Autism spectrum disorder
DEG	Differentially expressed gene
GO	Gene Ontology
KEGG	Kyoto Encyclopedia of Genes and Genomes
NGS	Next-generation sequencing
VPA	Valproic acid
GD	Gestational day
PND	Postnatal day
SDS	Sodium dodecyl sulfate
PPI	Protein–protein interaction
E/I	Excitation/inhibition
NMDA	N-methyl-D-aspartate
